# Optical Coherence Tomography Neuro-Toolbox for the Diagnosis and Management of Papilledema, Optic Disc Edema, and Pseudopapilledema

**DOI:** 10.1097/WNO.0000000000001078

**Published:** 2020-09-04

**Authors:** Patrick A. Sibony, Mark J. Kupersmith, Randy H. Kardon

**Affiliations:** Department Ophthalmology (PAS), State University of New York at Stony Brook, Stony Brook, New York; Departments of Neurology, Ophthalmology, Neurosurgery (MJK), Icahn School of Medicine at Mount Sinai and New York Eye and Ear Infirmary, New York, New York; Department of Ophthalmology and Visual Sciences (RHK), the University of Iowa, Iowa City, Iowa; and Center for the Prevention and Treatment of Visual Loss (RHK), Iowa City VA Health Care System, Iowa City, Iowa.

## Abstract

**Background:**

Distinguishing optic disc edema from pseudopapilledema is a common, sometimes challenging clinical problem. Advances in spectral-domain optical coherence tomography (SD-OCT) of the optic nerve head (ONH) has proven to be a cost effective, noninvasive, outpatient procedure that may help. At its core are tools that quantify the thickness of the retinal nerve fiber layer (RNFL) and ganglion cell–inner plexiform layer (GC-IPL). The SD-OCT also provides a set of tools that may be qualitatively interpreted in the same way that we read an MRI. They include the transverse axial, en face, and circular tomogram. Our goal is to describe a practical office-based set of tools using SD-OCT in the diagnosis and monitoring of papilledema, optic disc edema, and pseudopapilledema.

**Evidence Acquisition:**

Searches on PubMed were performed using combinations of the following key words: OCT, papilledema, pseudopapilledema, optic disc drusen, retinal folds (RF), and choroidal folds (CF).

**Results:**

The principal elements of SD-OCT analysis of the ONH are the RNFL and GC-IPL thickness; however, these metrics have limitations when swelling is severe. Qualitative interpretation of the transverse axial SD-OCT aids in assessing peripapillary shape that may help distinguish papilledema from pseudopapilledema, evaluate atypical optic neuropathies, diagnose shunt failures, and identify outer RF and CF. There is a consensus that the SD-OCT is the most sensitive way of identifying buried optic disc drusen. En face SD-OCT is especially effective at detecting peripapillary wrinkles and outer retinal creases, both of which are common and distinctive signs of optic disc edema that rule out pseudopapilledema. Mechanically stressing the ONH in the adducted eye position, in patients with papilledema, may expose folds and peripapillary deformations that may not be evident in primary position. We also discuss how to optimize the acquisition and registration of SD-OCT images.

**Conclusions:**

The SD-OCT is not a substitute for a complete history and a careful examination. It is, however, a convenient ancillary test that aids in the diagnosis and management of papilledema, optic disc edema, and pseudopapilledema. It is particularly helpful in monitoring changes over the course of time and distinguishing low-grade papilledema from buried drusen. The application of the SD-OCT toolbox depends on optimizing the acquisition of images, understanding its limitations, recognizing common artifacts, and accurately interpreting images in the context of both history and clinical findings.

## Abstract 2

Supplemental Digital Content is Available in the Text.

Distinguishing optic disc edema from pseudopapilledema is a common clinical problem that can challenge even the most experienced ophthalmologists (1). Differentiating optic disc edema from an optic disc anomaly is consequential because the recognition of pseudopapilledema obviates unnecessary and costly neurologic testing. In papilledema, an accurate diagnosis will avoid delays in treatment of potentially serious neurologic disorders.

Spectral-domain optical coherence tomography (SD-OCT), principally used in the management of maculopathies and atrophic optic neuropathies, was not specifically designed to evaluate optic disc edema, particularly when swelling is severe. Nonetheless, recent advances in SD-OCT imaging of the optic nerve head (ONH) have proven to be a cost effective, noninvasive, outpatient procedure that helps diagnose and manage optic disc edema and pseudopapilledema (1–4).

This review will focus on a toolbox of SD-OCT scanning modes that complement the retinal nerve fiber layer (RNFL) and ganglion cell–inner plexiform layer (GC-IPL) thickness in the diagnosis and management of an elevated nerve head. Our goal is to provide some practical guidelines with illustrations and cases for the performance and interpretation of the SD-OCT of the ONH.

## QUANTITATIVE SPECTRAL-DOMAIN OPTICAL COHERENCE TOMOGRAPHY ANALYSIS

Papilledema is a form of optic disc edema caused by an elevated intracranial pressure that extends into the perioptic subarachnoid space. Pressure, at the scleral flange and retrolaminar tissue of the optic nerve, has 2 principal effects as follows: i. axoplasmic stasis that distends prelaminar and peripapillary axons, thickens the peripapillary RNFL, and volumetrically expands the ONH and ii. anterior deformation of the lamina cribrosa and peripapillary tissues (toward the vitreous) (5). These effects are not exclusively pressure induced because optic disc edema in general may also be caused by ischemic, inflammatory, and neoplastic disorders of the optic nerve. SD-OCT can image and quantify each of the following structural effects: peripapillary shape deformations and increases in both the RNFL thickness and ONH volume (1–4).

### Retinal Nerve Fiber Layer in Optic Disc Edema

The defining characteristic of papilledema is distension of the papillary and peripapillary nerve fibers. With increasing disc edema, the normally transparent RNFL thickens, opacifies, and blurs the disc margin. As swelling increases, blood vessels become obscured, first at the margin and then on the surface of the optic disc. The Frisén grade is an ordinal scale that defines 6 stages (Grades 0–5) of swelling. SD-OCT measures the overall thickness of the RNFL with a resolution of 3–5 μm and a range of about 50–600 μm. The mean RNFL thickness correlates with the Frisén Grade and provides a continuous metric that reduces interobserver disagreement (6–11).

The Idiopathic Intracranial Hypertension Treatment Trial (IIHTT) found that 90% of the eyes with papilledema displayed an abnormal mean RNFL thickness (≥95% of normal controls) (12) consistent with a number of previously published reports using SD-OCT (6,8,10,13). Thickening of the RNFL is not specific to papilledema and may occur with any form of optic disc edema (14–17).

Registration of sequential circular or volume scans makes it possible to monitor the mean and regional RNFL thickness over the course of time (8,11,18). It is particularly effective at detecting small changes that may not be evident by ophthalmoscopy or disc photographs. The IIHTT SD-OCT substudy showed that there was a greater reduction in the mean RNFL thickness among the acetazolamide diet group than the placebo diet group at 6 months (11). This decrease in the mean RNFL thickness can take place within days, if not hours, after a spinal tap or shunt (19,20) Similar improvements were noted in the total retinal thickness (TRT) and ONH volume in eyes successfully treated in the IIHTT (11).

The central question in monitoring the mean RNFL thickness in optic disc edema over the course of time is determining what constitutes a clinically significant change. Much of the uncertainty stems from measurement variability between and within individuals and applying population metrics to an individual patient. This variability is increased in patients with papilledema, especially when swelling is severe (21–23). Although a decreasing mean RNFL thickness in a patient with papilledema is oftentimes a sign of improvement, it may also be an indicator of progressive axonal loss (24–26). This is a distinction that can only be made by simultaneously monitoring visual fields and thickness of the GC-IPL, providing that layer segmentation is accurate.

Some have suggested that small changes in the mean RNFL thickness are inconsequential and that monitoring the fundus and visual field are the principal factors that determine major therapeutic interventions (7). Without deprioritizing either, we suggest that when the mean RNFL thickness drops below 200 μm and the perimetric mean deviation is >−10.00 dB small changes in the mean RNFL thickness in the order of ±10–20 μm may also help provide early warnings of treatment failure. Such changes also help identify therapeutic noncompliance, provide guidance in adjusting medication dosages, and setting appropriate follow-up intervals. Conversely, the stability of the mean RNFL thickness, absent visual field changes, in patients with treated papilledema who for example develop unrelated migraines can be a reassuring indicator.

There are a number of caveats in the interpretation of the RNFL thickness many of which have been reviewed by Chen and Kardon, summarized in Table 1. The most important, with respect to papilledema, is that the segmentation algorithm may fail when papilledema is moderate to severe (mean RNFL > 200 μm) (1,6,12,29–31). Most devices display the segmentation line that defines the RNFL on the circular tomogram that should be checked for accuracy. There is some evidence to suggest that TRT and 3D segmentation algorithms may be less prone to segmentation failures than the mean RNFL thickness in severe papilledema. For the moment, both are limited to investigational studies (6,31–33).

**TABLE 1.** Limitations of the mean RNFL thickness

No normative values for children younger than 18 years
No normative adjustments for high myopes
Adults with “normal” (5%–95% of controls) mean RNFL thickness may still have optic disc edema Early or mild cases of papilledema (12). Anatomical variants with thin baseline RNFL thickness (7) Focal swelling (e.g., in NA-AION) Chronic papilledema
Thickening of the RNFL (>95% of controls) without optic disc edema (pseudopapilledema) Optic disc drusen especially in children (13,27,28) Other disorders of the optic nerve head and retina: gliosis, myelinated nerve fibers, retinal edema, hyperopia, and epiretinal membranes (29)
Segmentation failures especially in severe papilledema (1,11,12,29–31)
Artifacts (29) Low-signal strength ≤6 High myopia and high hyperopia Decentration of the circular tomogram Topographic variability of the RNFL bundles and thickness Movement artifacts Cyclotorsion Peripapillary atrophy

NA-AION, nonarteritic anterior ischemic optic neuropathy; RNFL, retinal nerve fiber layer.

Notwithstanding these limitations, the RNFL thickness is an effective tool at quantifying the magnitude of disc edema and monitoring changes over the course of time. However, the clinician must be cognizant of its strengths, limitations, and clinical context.

### Ganglion Cell–Inner Plexiform Layer in Optic Disc Edema

Thinning of the macular GC-IPL is a sign of neuronal loss that occurs in the atrophic stages of an optic neuropathy, such as glaucoma, optic neuritis, compressive optic neuropathy, and ischemic optic neuropathy. A decrease in the mean RNFL in optic disc edema due to axonal loss will cause thinning of the GC-IPL; whereas a decrease in the mean RNFL due restoration of axoplasmic flow should in theory at least spare the GC-IPL layer (1).

The problem is that commercial algorithms that use 2D imaging to measure the GC-IPL thickness may fail when there is significant ONH swelling. Artifactual thinning occurred in 20% of IIHTT study eyes (12,34). Scans with low-signal strength, outer retinal disorders, epiretinal membranes, and high myopia may also be associated with these segmentation artifacts (29,35,36). Segmentation failures can be reduced using custom 3D segmentation algorithms (12,31,37); however, these are not available in commercial devices. For now the use of GC-IPL in papilledema should be limited to patients with mild (≤Grade II) or chronic forms of papilledema but still need to be validated with a 10-2 visual field (38).

### Optic Nerve Head Volume

The SD-OCT measurement of ONH volume is another method of quantifying papilledema. This metric determines the volume of tissue between the internal limiting membrane and Bruch's membrane layer (BML) using a rectangular cube (6 × 6 mm) or a variety of concentric circular grids. ONH volume correlates with the mean RNFL thickness, TRT, and Frisén grade. It is increased in papilledema and other forms of optic disc edema and decreases with successful treatment as shown in the IIHTT (11,12,18,34). Although volume is a standard metric for the evaluation of the macula, its application to the ONH has been limited. It remains to be seen if ONH volume has any advantage over the RNFL thickness in the evaluation of optic disc edema. It has the potential to obviate the segmentation failures of the RNFL in severe papilledema (6,11,12,34).

## QUALITATIVE SPECTRAL-DOMAIN OPTICAL COHERENCE TOMOGRAPHY ANALYSIS

The qualitative assessment of the SD-OCT includes the cross-sectional transverse axial, radial scans, en face imaging, and circular tomogram. These scanning modes assess a variety of features that are not captured by the metrics of the SD-OCT of the ONH. They include, for example, shape deformations, pseudopapilledema, retinal folds (RF), choroidal folds (CF), retinal edema, inflammation, and hemorrhages among others.

### Transverse Axial: Shape and Displacement

Peripapillary shape refers to the configuration of the peripapillary BML on a 9-mm or 30° transverse axial SD-OCT. In selected cases, shape deformations may help evaluate and manage disorders that elevate the ONH.

The mechanical forces that anteriorly deform the posterior pole of the globe (toward the vitreous) include: the intraocular pressure, cerebrospinal fluid (CSF) pressure (sometimes also expressed as the translaminar pressure difference or gradient), retrolaminar tissue pressure, and the orbital tissue pressure. An imbalance between these forces may reshape the globe. The effect is modulated by the structural geometry of the ONH and compliance of the load-bearing structures (sclera, lamina, and dura) (39–42). An increase in the CSF or orbital tissue pressure, a decrease in intraocular pressure, stiffening of the optic nerve, or the optic nerve sheath may cause anterior shape deformations. These deformations can help distinguish optic disc edema from pseudopapilledema.

**FIG. 1.**
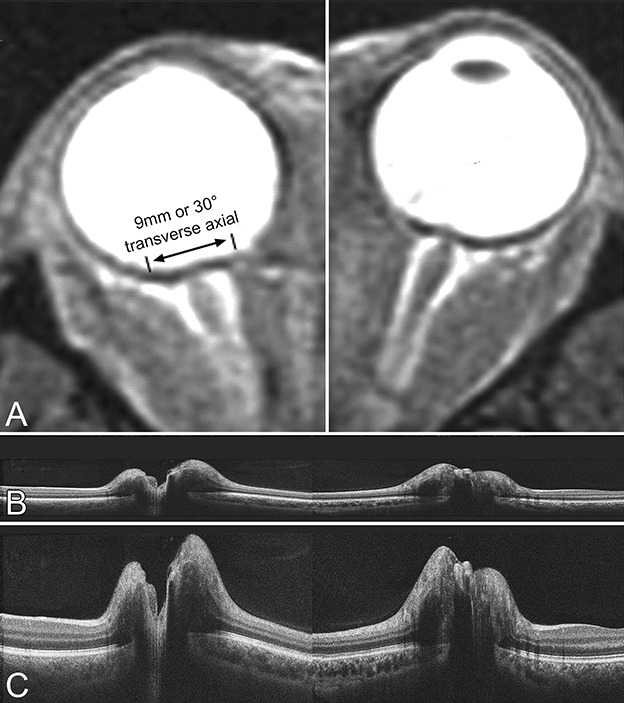
**A**. MRI showing distended optic nerve sheaths with flattening of the globe. Corresponding (**B**) unstretched transverse axial OCT and (**C**) 3× vertically scaled transverse axial OCT. OCT, optical coherence tomography.

Experimentally, a change in the translaminar pressure difference causes an anterior or posterior displacement of the optic disc depending on the direction of the gradient (43,44). Clinically, elevation of the CSF pressure flattens the globe; a deformation that can be imaged on MRI (45) (Fig. 1A) or B-scan ultrasonography (46,47). The transverse axial SD-OCT of the ONH and peripapillary tissues can also image flattening of the globe providing a high-resolution, cost effective, alternative to the MRI and ultrasound (Fig. 1B, C) (19,48,49).

Several morphometric approaches have been used to gauge peripapillary deformations and displacements using SD-OCT in patients with intracranial hypertension and papilledema. They include angular deflections of the BML (48,50,51), displacements of BM opening (19) or anterior laminar surface depth (52–55), and geometric morphometric shape analysis (19,49,56,57). Each has limitations. For example, it is not always possible to identify a undisplaced peripheral baseline of BML to measure angular deflections in severe cases of optic disc edema. Because of limited penetrance of near-infrared light of the SD-OCT, the anterior laminar surface is not always visible even with adaptively enhanced swept-source OCT (53). Geometric morphometrics is robust and sensitive but impractical as an office-based clinical tool (49).

Nonetheless, these studies have shown that shape or displacement of BML can be used as an indirect approximation of intracranial pressure especially for monitoring changes over the course of time. Patients with papilledema have a peripapillary shape and anterior laminar surface that is anteriorly deformed or displaced compared with normals, pseudopapilledema, and nonarteritic anterior ischemic optic neuropathy (NA-AION). Moreover, interventions that lower the CSF pressure (e.g., after lumbar puncture, CSF shunts, acetazolamide treatment, and optic nerve sheath fenestrations) cause a relative posterior shape deformation or displacement (19,48–57). Although these anterior deformations are most frequently seen in patients with intracranial hypertension and papilledema, they may also occur, less commonly, in optic nerve sheath meningiomas (16), NA-AION (58), and optic neuritis (48).

**FIG. 2.**
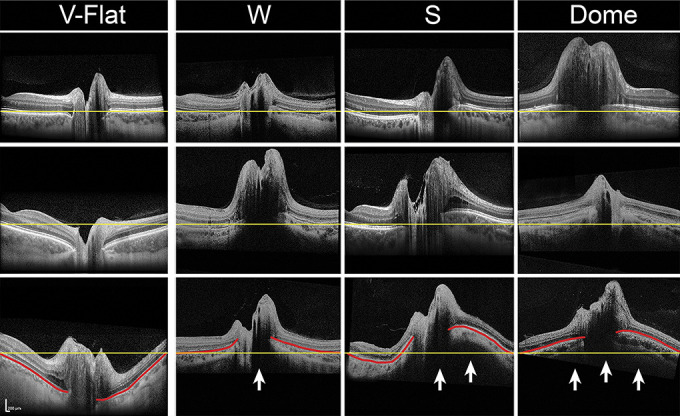
Peripapillary shape patterns on transverse axial OCT (30°, 3× vertically scaled; left side of each inset is temporal to the disc). Shapes are defined by the configuration of Bruch's membrane layer (BML, illustrated in bottom row with a *red line*) relative to horizontal *yellow line* that connects the peripheral ends of BML. The most frequent pattern (V-Flat column) is a continuum that falls between an F (flat)-shape and V-shape that lies at or entirely below the horizontal reference line. There are 3, sometimes overlapping, shape patterns associated with anterior deformation or displacement (columns WSDome). The W-shape consists of symmetrical anterior deflection (toward the vitreous) of the inner margins of the BML. The S-shape is anteriorly displaced toward the vitreous nasally above the reference line, and posteriorly displaced temporally, below the horizontal reference that tilts Bruch's membrane opening. The D or dome shape is a broad-based symmetrical, anterior displacement of the peripapillary BML above the horizontal reference line. The *white arrows* depict the forces acting on the optic nerve head that presumably give rise to these shape patterns. OCT, optical coherence tomography.

In the clinical setting, peripapillary shape is assessed qualitatively with the transverse axial SD-OCT. We have identified several common shape patterns (Fig. 2). The most frequent is a continuum between a flat (F)-shape and V-shape (Fig. 2, V-Flat column) that occurs in normal healthy subjects, pseudopapilledema (with or without optic disc drusen [ODD]), NA-AION, and papillitis. By contrast, anterior deformations and displacements give rise to several distinctive patterns that we describe as a 1) W-shape, 2) S-shape or 3) D-shape or Dome shape (Fig. 2, columns W, S, and D). These occur most frequently in patients with intracranial hypertension, papilledema, and optic nerve sheath meningiomas and rarely in patients with ocular hypotension, optic neuritis, and NA-AION. The absence of anterior shape deformation or displacement in a patient with optic disc edema does not necessarily exclude intracranial hypertension or optic nerve sheath meningiomas (16). Approximately one-third of patients with papilledema may have a “normal” V-F shape. Without a baseline for comparison, it is not possible to determine whether a V-F shape in papilledema is in fact mildly displaced anteriorly relative to its shape when the pressure is normal (Fig. 6).

**FIG. 3.**
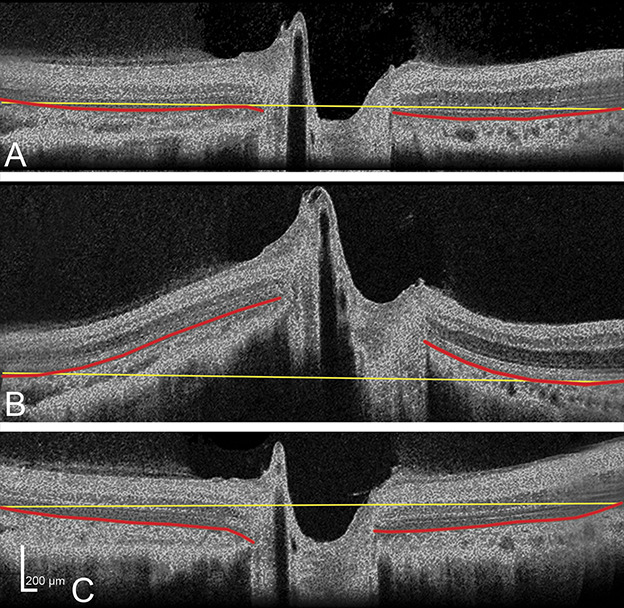
Transverse axial (30°, 3× vertical stretch) OCT of a 32-year-old man with a history of hydrocephalus and ventriculoperitoneal shunt. **A**. Baseline OCT with a functioning shunt has a relatively flat or F-shape with optic atrophy (mean RNFL thickness 45 μm). **B**. Six months later, shunt failure is associated with an anterior deformation (D-shape) and a slight increase in the mean RNFL thickness to 54 μm. **C**. One month after shunt revision showing normal V-shape and the mean RNFL thickness of 46 μm. Shape changes are independent of the degree of papilledema and particularly helpful in patients with optic atrophy and intracranial hypertension. OCT, optical coherence tomography; RNFL, retinal nerve fiber layer. Red line delineates the shape of Bruch's membrane layer (BML). Yellow line is a reference line that joins the peripheral margins of BML.

**FIG. 4.**
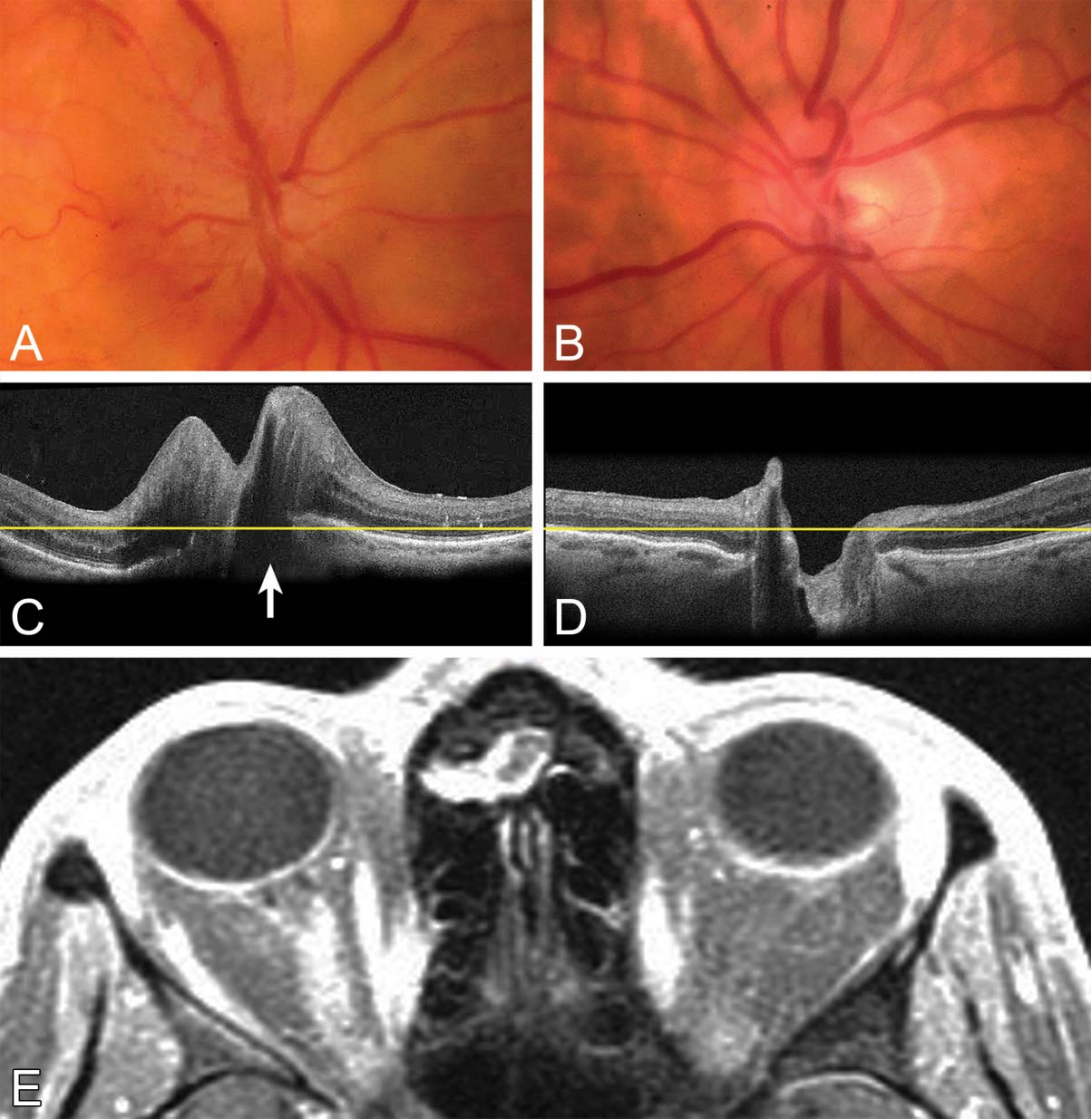
A 66-year-old diabetic woman with sudden painless vision loss, 20/40, an afferent pupillary defect, a nasal step, and unilateral optic disc edema in the right eye (**A**). The left eye was normal with cup-to-disc ratio of 0.2 (**B**). A presumptive diagnosis of nonarteritic anterior ischemic optic neuropathy was made. Because the transverse axial OCT showed anterior deformation with a W-shape in the right eye (**C**) vs V-shape in the left eye (**D**), we obtained an MRI (**E**) that showed a presumed optic nerve sheath meningioma. OCT, optical coherence tomography. Yellow line is a reference that joins the peripheral margins of Bruch's membrane layer. The white arrow highlights the anterior displacement or BML relative to the yellow reference line.

In selected cases, qualitative peripapillary shape assessments may help as follows: i. distinguish pseudopapilledema from papilledema (Fig. 12); ii. identify shunt failures, particularly in patients with optic atrophy (Fig. 3); iii. evaluate atypical anterior optic neuropathies (Fig. 4); and iv. corroborate small but clinically significant changes in the mean RNFL thickness in patients with intracranial hypertension (Fig. 3).

#### Image Acquisition and Interpretation

**FIG. 5.**
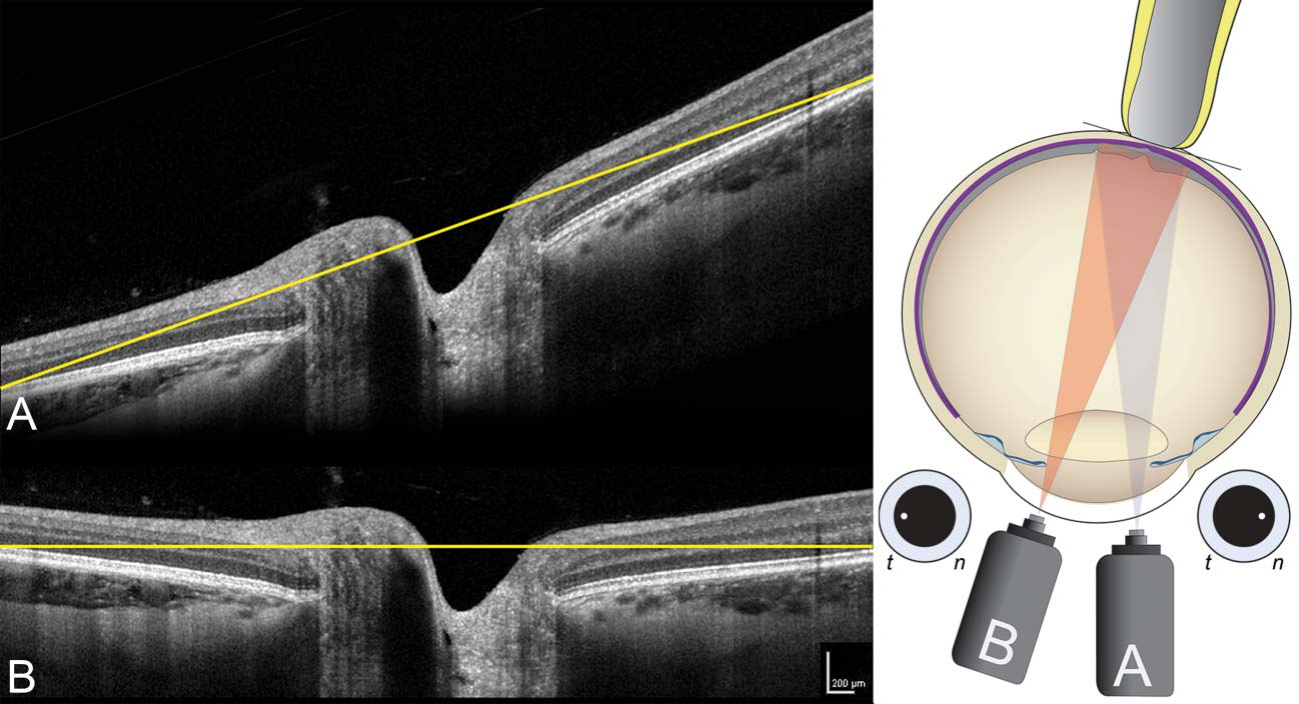
Tilted image artifact (**A**) vs horizontal flat (**B**) image. To obtain a symmetrical, flat image the camera needs to be positioned approximately 12° temporal to the pupillary axis, aimed nasally at the posterior pole so that the scan is symmetrically positioned over the optic disc (camera **B**). The precise position of the scanning beam on the pupillary axis may vary but can be adjusted using the preview screen.

**FIG. 6.**
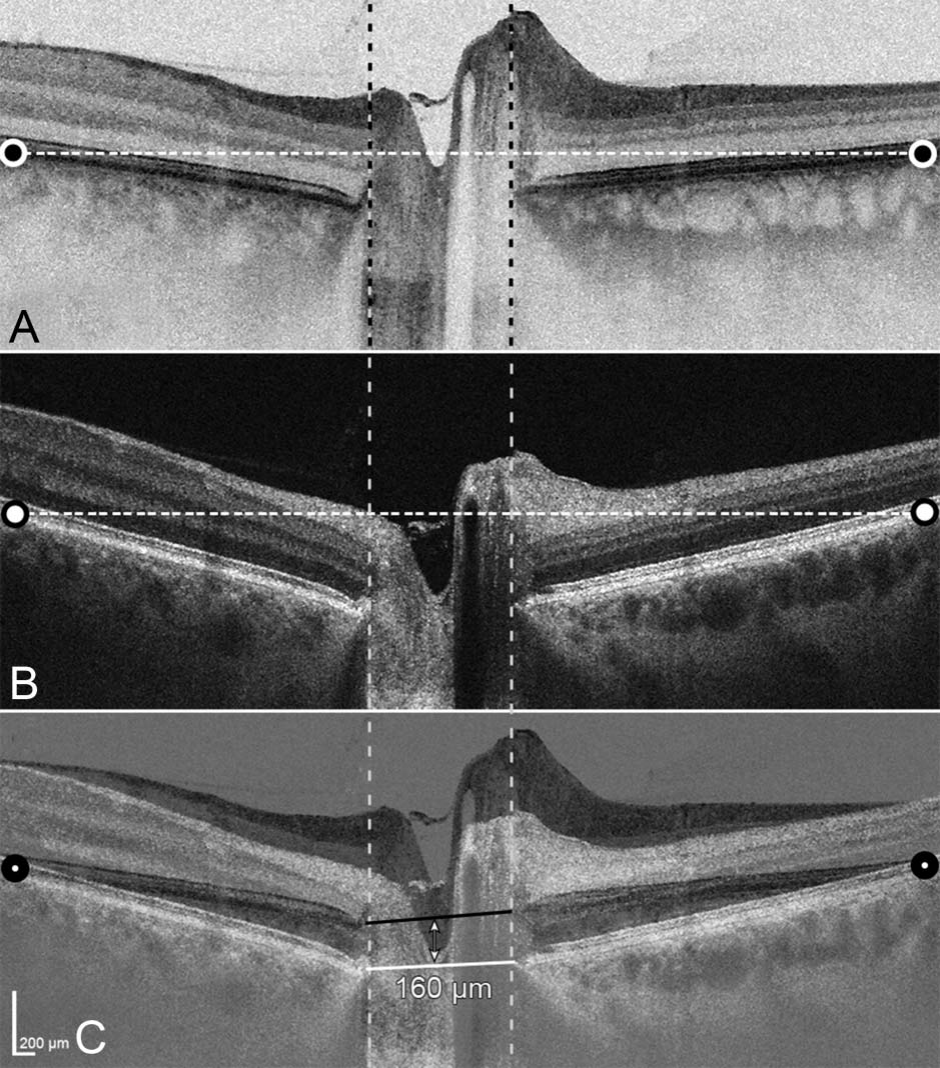
Registration of two 30° transverse axials (3× vertically scaled), from the same patient obtained at (**A**) baseline and (**B**) 6 weeks later on acetazolamide. The landmarks on the peripheral Bruch's membrane layer (BML, *black circles* in **A**) and (*white circles* in **B**) are superimposed in image (**C**) so that the Bruch's membrane opening (BMO) is aligned with the vertical *dashed lines*. At baseline, the mean retinal nerve fiber layer (RNFL) thickness was 118 μm with a mild V-shaped configuration of BML relative to the horizontal dotted reference line (**A**). One month later, on treatment there is a decrease in the mean RNFL at 96 μm. The BMO was displaced 160 µm posteriorly and the V-shape of the BML is steeper (**B**). Comparison of sequential transverse axials can be performed in the outpatient setting by superimposing tracings of printed reports on a light box.

Transverse axial images are best acquired as a high-resolution, wide angle (9 mm or 30°) image with enhanced depth imaging (EDI). The default transverse axial images from many of the commercial SD-OCT devices are vertically stretched indicated in the accompanying scale bars (Fig. 1B, C). Increasing the vertical scale is a useful feature for assessing intraretinal details and shape (59). Avoid the tilted image caused by an off-axis scanning beam relative to the ONH (Fig. 5) (49). This is a common artifact that distorts the ONH shape and affects the mean RNFL thickness (60). The registration method used to compare shape changes over the course of time or between eyes is shown in Figure 6.

### Transverse Axial: Pseudopapilledema

#### Pseudopapilledema With Optic Disc Drusen

The misidentification of ODD usually occurs in the young when ODD are small, buried, and concealed from view. They simulate low-grade papilledema, chronic papilledema, or resolved papilledema because the optic discs are elevated with indistinct disc margins even when the drusen load (in size and number) is small. Ophthalmoscopic signs such as vessel branching anomalies, the absence of venous engorgement, the lack of disc vessel obscuration, and clearly defined peripapillary nerve fibers all support, but do not definitively confirm, the diagnosis of ODD (61). The superficial refractile bodies of chronic papilledema are distinctive pinpoint, glistening, yellow deposits that have been misdiagnosed as ODD (62). Rarely, complications of ODD such as peripapillary subretinal hemorrhages or vascular occlusions may confound the diagnosis. Most confusing are cases with both papilledema and ODD (Fig. 12, see discussion below) (61).

Ancillary testing can help differentiate pseudopapilledema from papilledema but each has limitations. Small changes in papilledema can be difficult to discern, even with serial disc photographs. Late staining of the ONH in pseudopapilledema can be mistaken for disc leakage in papilledema. Computed axial tomography of head to detect calcified ODD is inconvenient and exposes the patient to radiation. Autofluorescence imaging may miss small buried drusen. Ultrasound, long considered the gold standard, may also fail to detect small buried drusen (13,63).

**FIG. 7.**
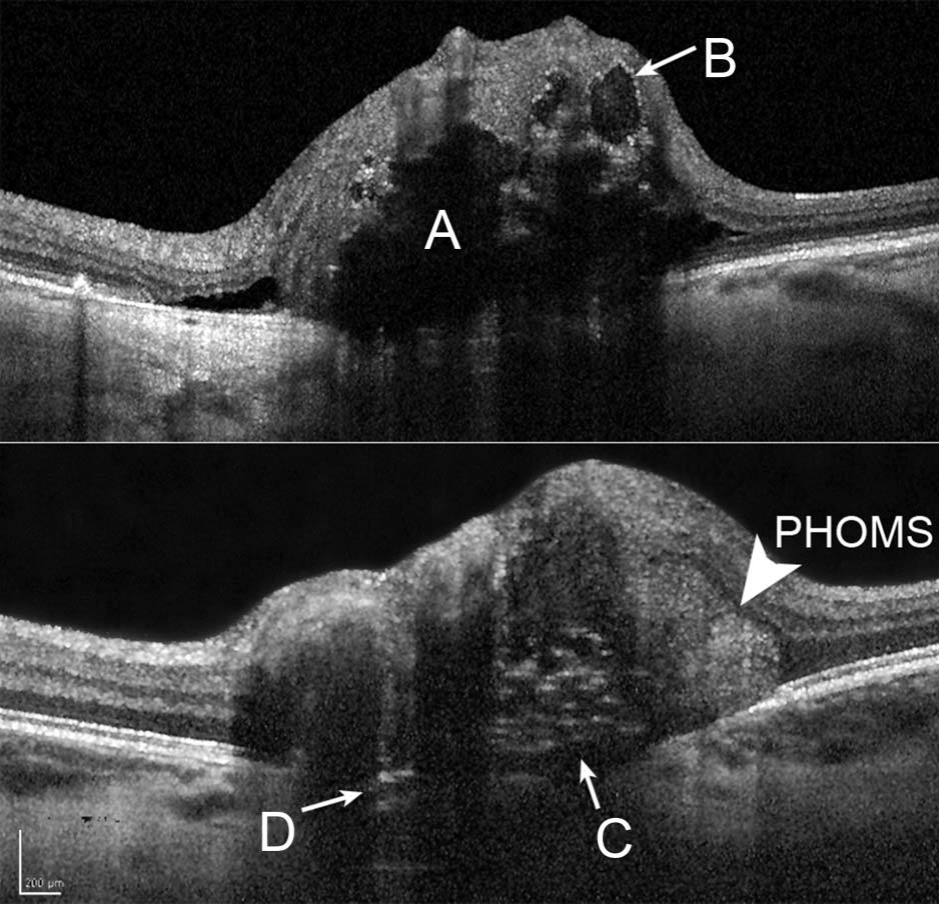
Salient features of optic disc drusen on the transverse axial includes: (**A**) signal poor core occasionally associated with a (**B**) hyperreflective cap or (**C**) multiple small hyperreflective aggregates within a signal poor core. Small horizontal bands are also shown in (**D**). Peripapillary hyperreflective ovoid mass like structure (PHOMS, *white arrowhead*) are not drusen.

There is an emerging consensus that the SD-OCT is the most sensitive way of detecting ODD (64–66). The Optic Disc Drusen Studies Consortium (66) has proposed criteria for the SD-OCT diagnosis of ODD that consists of a defined, prelaminar, signal poor lesion sometimes associated with a hyperreflective cap or multiple small hyperreflective aggregates within a signal poor core. These findings are occasionally accompanied by small horizontal hyperreflective bands (Fig. 7). Isolated bands have been observed in about 14% of healthy subjects with normal optic discs suggesting that drusen may be more common than previously known (67). There is some evidence, based on long-term follow-up over a 5-year period, to suggest that these hyperreflective lines may represent ODD precursors (68).

A peripapillary hyperreflective ovoid mass like structure (PHOMS, Fig. 7) is an SD-OCT finding in some patients with ODD (69). Early reports on the OCT in ODD have asserted that these are uncalcified precursors of drusen (13,28,70–76). The consortium has argued against this view suggesting instead that these are distended prelaminar axons bulging into the peripapillary retina. Histopathologically, PHOMSs in ODD are uncalcified. Identical structures, shown histopathologically and by SD-OCT, occur in papilledema (77), pseudopapilledema without drusen (78) (see below), and other forms of optic disc edema. Finally, unlike the permanence of ODD, PHOMS dissipate when optic disc edema resolves and is absent in advanced cases of ODD with optic atrophy (79–81).

#### Image Acquisition and Interpretation

ODD can be overlooked with a poor quality OCT. The ODD Studies Consortium recommends that the SD-OCT imaging mode include densely sampled (B-scan intervals of <30 μm), high-resolution transverse axial images with EDI that spans the diameter of the ONH. They also recommend 6-line radial scans, RNFL thickness, and macular volume scans for GC-IPL (69).

#### Pseudopapilledema Without Drusen

**FIG. 8.**
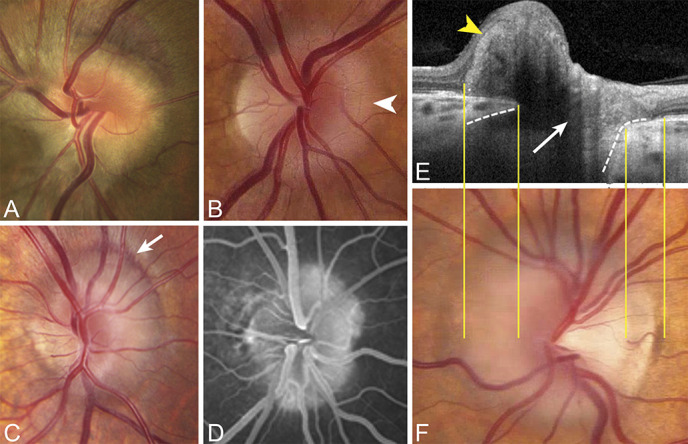
Pseudopapilledema without drusen. **A**. Tilted optic disc syndrome with significant rotation, an oval shape, situs inversus; (**B**) ophthalmoscopic features of the myopic obliquely inserted disc (MOID) consist of a pale nasal C-shaped halo (*white arrowhead*), nasal elevation, obscuration of the nasal disc margin, and little if any rotation. **C**. A slightly rotated MOID with an asymptomatic peripapillary subretinal hemorrhage (*white arrow*); (**D**) late staining of the nasal disc on fluorescein angiography sometimes seen in a MOID; (**E**, **F**) salient optical coherence tomography features of MOID with corresponding fundus photograph showing nasal elevation, (**E**, **F**) oblique entry of the optic nerve (*white arrow*), peripapillary hypopigmentation temporally, and peripapillary hyperreflective ovoid mass like structure (*yellow arrowhead*) that corresponds to the C-shaped halo nasally in the photograph. Yellow lines in (**E**, **F**) show corresponding locations in the OCT image and the optic disc photo.

The tilted optic disc, in its most typical form, is a congenital anomaly characterized by oblique entry of the optic nerve, superonasal elevation, situs inversus, and a rotated oval or D-shaped disc. These features in combination with inferior chorioretinal thinning and superior temporal visual field loss constitute the tilted disc syndrome (TDS) (82). With some exceptions (83), these optic discs are so dysmorphic that they usually are not confused with papilledema (Fig. 8A).

There is a form of pseudopapilledema without drusen sometimes also referred to as tilted optic disc, myopic disc, or congenital disc anomaly. We propose to distinguish this particular anomaly from TDS by referring to this form as a “myopic, obliquely inserted disc” (MOID). The salient funduscopic and OCT features are described in Figure 8B–F. In our experience, this is the single most common reason that patients are referred to “rule out” papilledema or ODD. These optic discs are sometimes confused with papilledema because they are frequently associated with a PHOMS that elevates the nasal disc and obscures the nasal disc margin with a pale C-shaped halo seen funduscopically. Similar features have been previously described by Pichi et al (78) in children with TDS. What they called a “dome-shaped hyperreflective structure” is identical to a PHOMS described in ODD and optic disc edema. The rare occurrence in a MOID of an asymptomatic spontaneously acquired peripapillary subretinal hemorrhage (Fig. 8C) or the late staining nasally on fluorescein angiography can be particularly confounding (Fig. 8D) (84). It is uncertain, if TDS and MOID represent the opposite ends of a continuum or if these are 2 distinct entities with overlapping features (78,84).

Some have asserted that the PHOMSs in patients with MOIDs are uncalcified ODD (70,73). The evidence against this view was discussed in the preceding section (78,80). Irrespective of nomenclature, a PHOMS seems to be a common nonspecific structural feature on SD-OCT in patients with ODD, optic disc edema, and optic disc anomalies, such as MOID and TDS.

### Transverse Axial: Other Features

**FIG. 9.**
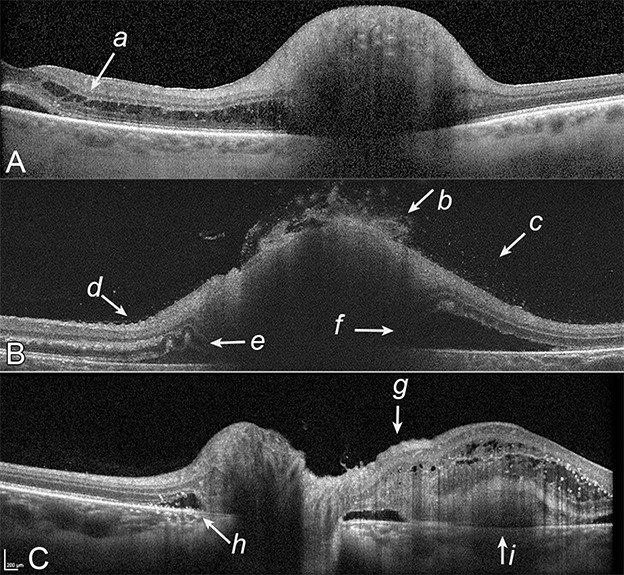
Transverse axial images: (**A**) Nonarteritic anterior ischemic optic neuropathy with peripapillary subretinal fluid and macular edema, serous pigment epithelial detachment (a); (**B**) neuroretinitis (85) with (b) epipapillary inflammatory infiltrates, (c) vitreous cells, (d) peripapillary wrinkles and inner retinal folds, (e) outer retinal creases, and (f) peripapillary subretinal fluid; (**C**) Papilledema with (h) peripapillary fluid, (i) choroidal neovascular membrane and subretinal hemorrhage, and (g) inner retinal folds.

The transverse axial SD-OCT can aid in distinguishing NA-AION from neuroretinitis (85) (Fig. 9). It can also help distinguish the vision loss of an optic neuropathy from a maculopathy. This would include findings such as epiretinal membranes, maculopathies, choroidal neovascular membranes, and pigment epithelial detachments.

### En Face Spectral-Domain Optical Coherence Tomography: Wrinkles, Folds, and Creases in Papilledema

The wrinkles, folds, and creases that occur in papilledema are structural consequences of stress and strain of intracranial pressure on the ONH and the load-bearing structures (sclera and lamina cribrosa). Clinically, the recognition of folds strongly supports the diagnosis of optic disc edema and effectively eliminates pseudopapilledema from consideration. They are particularly helpful in distinguishing low-grade papilledema from pseudopapilledema (86,87).

**FIG. 10.**
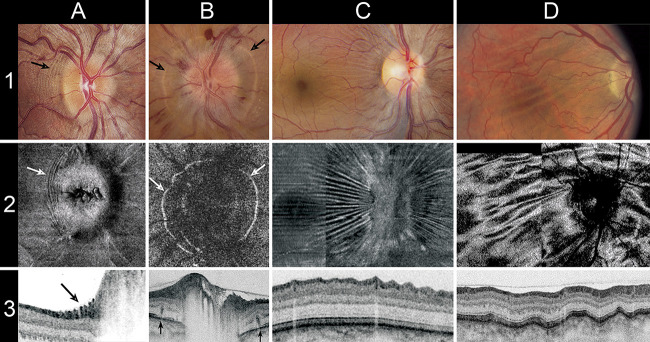
Four types of folds in papilledema (columns **A**–**D**) shown with photographs (row 1), en face OCT (row 2), and cross-sectional OCT (row 3). **A**. Peripapillary wrinkles (PPW) or Paton's folds are closely spaced (∼110 μm) undulations temporal to the optic nerve head (ONH) in the RNFL (*arrows*). The pattern is usually concentric to the optic disc or may spiral toward the macula. PPW can occur in any type of optic disc edema and best imaged with en face OCT (A2). **B**. Peripapillary outer retinal folds or creases are widely spaced (∼300 to 450 μm) and spare the RNFL. Funduscopically, they are commonly referred to as “high-water marks” (B1, *black arrows*). Early on they may be associated with subretinal fluid. As the fluid resorbs it leaves behind a deeply furrowed self-contacting crease in the outer retina that appears en face as circumpapillary ring (B2, *white arrow*) and a vertical line on the transverse axial (B3, *black arrow*). **C**. Inner retina folds (∼230 μm) spare the choroid. In papilledema they tend to radiate out from the ONH (C1, 2) or consist of horizontal folds in the papillomacular bundle. They are best imaged with en face OCT (C2) or perpendicularly oriented line scans (C3) or circle tomogram. **D**. Choroidal folds are widely spaced (∼530 μm) full-thickness folds distributed horizontally or obliquely across the posterior pole (D1, 2) and funduscopically associated with the RPE striations (D1). In patients with intracranial hypertension, choroidal folds correlate with intracranial pressure and anterior shape deformation of the peripapillary tissues. They are best imaged with a perpendicularly oriented cross-sectional OCT (D3) or circle tomogram. OCT, optical coherence tomography. RPE, retinal pigment epithelial.

Folds are common in patients with papilledema and other causes of disc edema (77,86–89). Based on the IIHTT, folds were detected with SD-OCT in at least one eye in 73% of patients. SD-OCT was more sensitive in detecting folds than fundus photographs (43%). There were 4 types of folds as follows: peripapillary wrinkles (PPW, 46%), inner RF (IRF, 47%), outer retinal folds and creases (ORF, 20%), and CF (10%). Each has distinctive characteristics with respect to location, pattern, spatial wavelength (λ), temporal course, and underlying biomechanics (86,87). The SD-OCT and photographic features of each type of fold are shown in Figure 10.

**FIG. 11.**
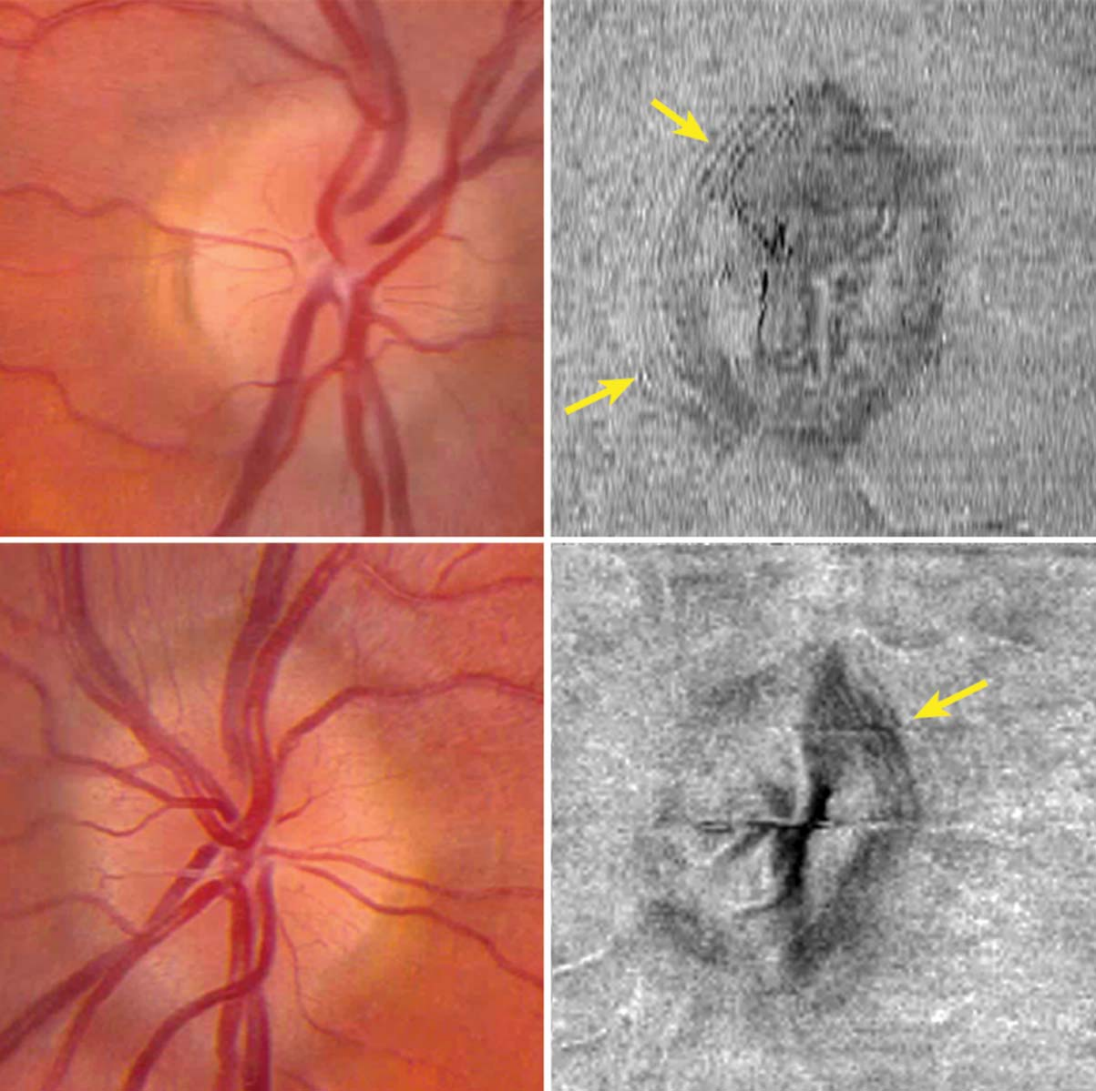
A 31-year-old woman with occasional headaches for 2 years, body mass index 30.5. Examination was otherwise normal. No drusen by B-scan or OCT; the mean RNFL thickness was 106 μm in the right eye, 101 μm in the left eye; no anterior deformation; en face OCT showed peripapillary wrinkles temporally in both eyes (*yellow arrows*). MRI/MRV was normal. Cerebrospinal fluid pressure was 33 cm. Some patients adapt to long-standing intracranial hypertension with minimal swelling, preservation of vision, in effect a chronic “compensated” papilledema. OCT, optical coherence tomography; RNFL, retinal nerve fiber layer. MRV, Magnetic resonance venography.

PPW, ORF and creases (high-water marks), and IRF usually resolve over a period of months to a year in papilledema and more rapidly in NA-AION and neuroretinitis. On occasion, PPW can persist for years, long after the optic disc edema resolves and thus may provide a telltale sign of mild, resolving, or chronic papilledema (Fig. 11) (85,86,88,89). Although the amplitude of CF may decrease with time, they are distinctively persistent long after the papilledema resolves. We have followed patients with CF for many years, in one case for 15 years without resolution. Patients with “idiopathic” acquired hyperopia and CF even in the absence of optic disc edema should be evaluated for intracranial hypertension (90). This presentation is sometimes seen in patients with sleep apnea. CF with mild disc edema have also been described in spaceflight-associated neuro-ocular syndrome (91,92).

**FIG. 12.**
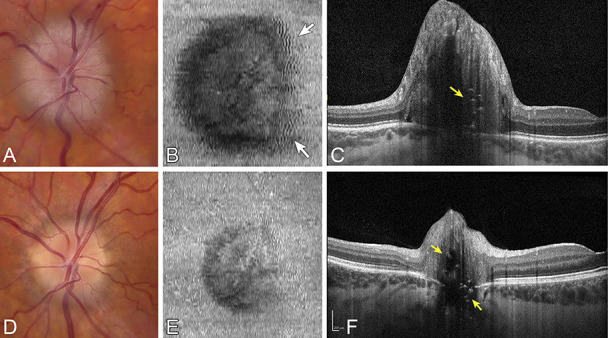
A 16-year-old girl with optic disc drusen and papilledema (98). Baseline examination of left eye (**A**–**C**) showed an elevated optic nerve head and optic disc drusen confirmed by ultrasound, autofluorescence, and OCT (**C**, *yellow arrow*). However, the patient has peripapillary wrinkles (PPW) on the en face OCT (**B**, *white arrows*), and transverse axial showed an anterior (S-shaped) deformation (**C**) with a mean RNFL thickness of 373 μm. MRI revealed hydrocephalus due to an aqueductal stenosis. She was treated with an endoscopic third ventriculostomy. Six months later, the mean RNFL thickness decreased to 84 μm and OCT showed a reduction in the PPW (**D**, **E**) and a V-shape (**F**). Optic disc drusen are more visible (**F**, *yellow arrows*) on transverse axial OCT. The right eye (not shown) was similar in all respects. OCT, optical coherence tomography; RNFL, retinal nerve fiber layer.

There is a widely held clinical precept that folds do not occur in ODD and that their presence distinguishes optic disc edema from pseudopapilledema. There have been several reports of ODD with CF, but it is not possible to establish a causal connection based on these 3 cases alone (93–95). A number of studies on the SD-OCT of pseudopapilledema with and without drusen have been reported but none specifically commented on the presence of folds (28,64,65,69,96,97). We recently studied the en face and axial SD-OCTs of 102 patients with ODD for PPW, ORF, IRF, and CF (98). One or more types of folds were present in 11 of the 102 (11%) patients with ODD, all of whom had a coexistent optic disc edema due to papilledema (6/11), NA-AION (4/11), or uveitic disc edema (1/11). There were 2 additional patients (2%) with ODD who had PPW without optic disc edema. These findings indicate that the occurrence of folds in ODD is frequently associated with a complication of ODD, such as NA-AION or an unrelated papilledema (Figs. 11, 12). The cause of PPW in 2 of the patients (without optic disc edema) is unknown (86). We cannot exclude a previous subclinical bout of NA-AION or papilledema in either of these patients.

The coexistence of ODD and papilledema (99,100) has generally been assumed to be coincidental (Fig. 12) (2). However, 2 recent studies found a high frequency in adults and children with both ODD and papilledema at 19% (75) and 48% (76); far greater than the estimated prevalence of ODD at 0.3%–2.4% in healthy subjects. Although both studies used multiple modalities, the OCT criteria used to diagnose ODD may have overestimated its frequency by relying on low-resolution Time-domain OCT (with limited penetrance) or basing the diagnosis on PHOMS alone (75,76). Neither study met the recommended criteria for the SD-OCT diagnosis of ODD, and thus it remains uncertain whether there is an association between papilledema and ODD. Nonetheless, we have seen enough of these cases to remain open to the possibility pending a large case series that use the consortium recommendations (66) for the SD-OCT diagnosis of ODD.

#### Image Acquisition and Interpretation

**FIG. 13.**
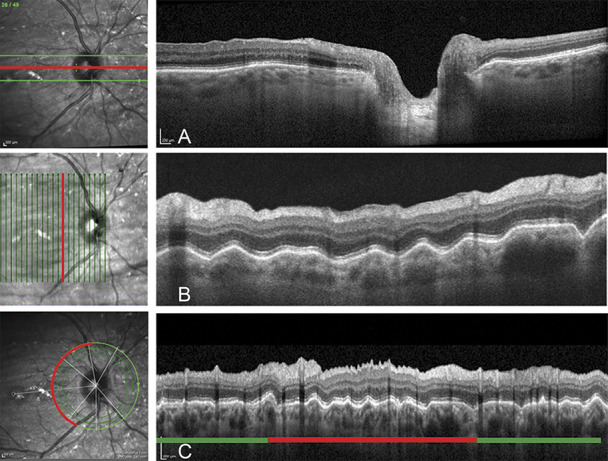
Transverse axial, sagittal, and circular optical coherence tomography from the same subject with horizontal choroidal and inner retinal folds. Folds parallel to the transverse axial scan are not visible (**A**). Vertical scans perpendicular to the folds clearly display folds (**B**). Circular tomogram (**C**) will sometimes include a segment of the circumference that perpendicularly intersects with folds if they cross the circular scan. Red lines in the scanning laser images (left column) shows the relative location of the corresponding cross sectional. **B**. Scans in the right column. Green lines show location and orientation of the cross sectional B scans.

Proper screening for folds using en face SD-OCT requires optimal positioning of the segmentation lines. The offset must straddle the surface of the retina, and the slab must be thin enough to capture the peaks and troughs of the folds. Some devices require closely spaced B-scan intervals (≤60 μm apart) to obtain en face images. In addition, transverse axial SD-OCT scans must be oriented perpendicular to the fold to be seen (Fig. 13).

### Stress Testing the Optic Nerve Head

**FIG. 14.**
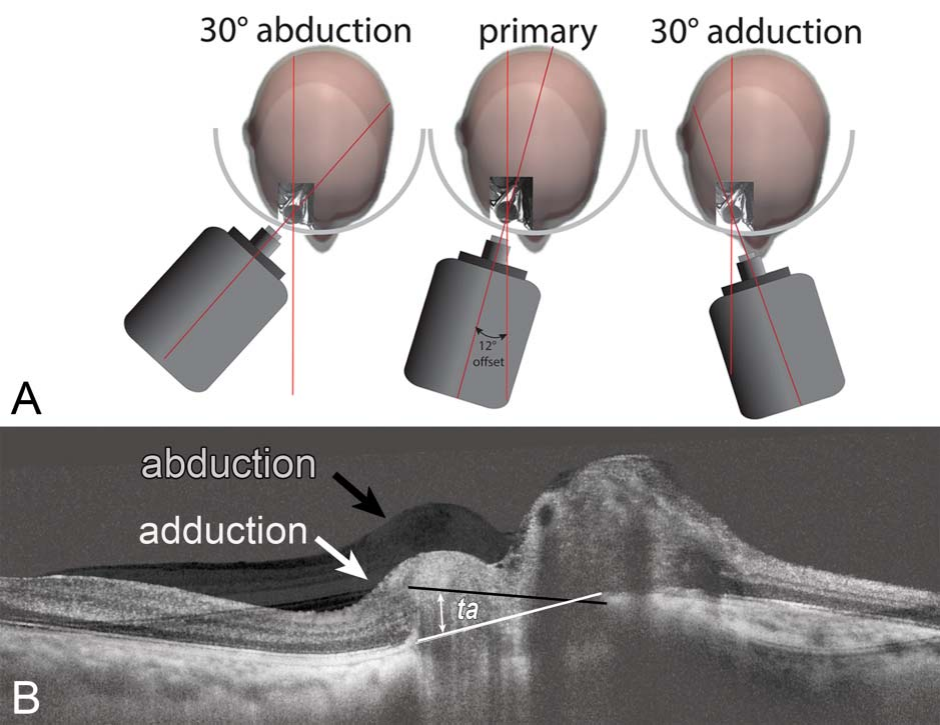
Transverse axial optical coherence tomography (vertical stretch of 1.4×) acquired in primary position, 30° abduction and 30° adduction (**A**). The images obtained in abduction (*black*) and adduction (*white*) are superimposed in (**B**). The 2 lines that connect the naso–temporal margins of the Bruch's membrane opening obtained in abduction (*black line*) and adduction (*white line*) intersect to form the tilt angle (ta). In adduction the temporal side of the Bruch's membrane opening is posteriorly displaced (away from the vitreous, *white line*) relative to the nasal side. In abduction displacement is reversed. Folds can be seen on the temporal slope of the optic nerve head in adduction only (*white arrow*, in **B**).

Ocular ductions induce small alternating “seesaw” deformations of the ONH and peripapillary retina in normal subjects, NA-AION, and ODD. In adduction, the temporal margin of the BML is displaced posteriorly (away from the vitreous), the nasal side moves anteriorly, and the reverse occurs in abduction (101–103). In adduction, stretching the optic nerve sheath tethers the peripapillary tissues temporal to the ONH (104). The magnitude of these seesaw deformations can be measured as a “tilt angle” (Fig. 14). The magnitude of this angle using 1.4× vertically scaled images in normal subjects, NA-AION, and ODD is small, in the order of 2°–4° (105). Although the pattern of deformation in papilledema is the same, the magnitude of the tilt angle is exaggerated (101) (8°–20°) (105) presumably because the mechanical load of the optic nerve sheath is augmented by gaze-induced shifts of the CSF and hydraulic stiffening of the optic nerve and the sheath. Please see **Supplemental Digital Content 1** (see **GIF image**, http://links.lww.com/WNO/A440), which illustrates the seesaw deformations induced by ocular ductions in normal subjects, NA-AION, ODD, and papilledema (101).

**FIG. 15.**
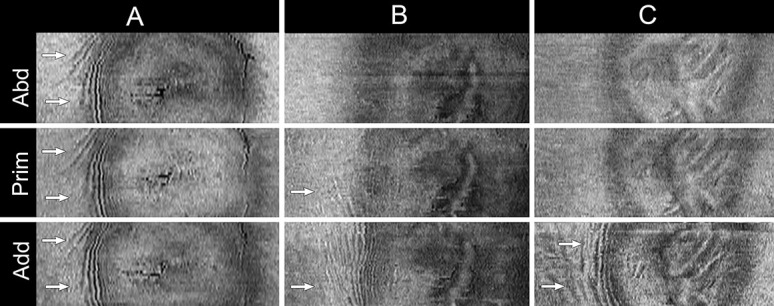
En face optical coherence tomographies at the vitreoretinal interface obtained in primary position (prim), 30° abduction (abd), and 30° adduction (add). Three patterns are displayed. **A**. Peripapillary wrinkles (PPW) in primary position that are not affected by changes in eye position; (**B**) PPW in primary that become more evident in adduction and absent in abduction; (**C**) an example of PPW that are absent in primary and visible only when the eye is adducted. The *white arrows* in each case highlight the area of folds temporal to the optic disc.

The mechanical forces on the ONH induced by ocular ductions can also affect the pattern and distribution of PPW (105). As force increases, the amplitude and the area of the folds increase, and thus PPW located temporally become more evident when the eye is adducted 30° (Fig. 15B). Sometimes, folds that are absent in primary position are only visible in adduction, particularly with large tilt angles (Fig. 15C). This does not seem to be the case in normal subjects and ODD with mild elevation (105). Examining the eye in adduction increases the sensitivity of detecting folds that may help to differentiate papilledema from pseudopapilledema. Large disparities in shape between primary position and adduction, that is, with large tilt angles (Fig. 14B) can also be a sign of intracranial hypertension and optic nerve sheath meningiomas.

En face examination for folds should be considered in all presumed cases of pseudopapilledema even those with ODD. If absent in primary, the patient should be examined in adduction. Although not as sensitive as SD-OCT, the same maneuver can be used with fundus photographs obtained with the eye in adduction.

### Summary of the Spectral-Domain Optical Coherence Tomography Toolbox for Disc Edema

In summary, the SD-OCT supplements but does not replace the ophthalmic examination. It helps distinguish low-grade papilledema from pseudopapilledema and aids in monitoring changes. At its core are the quantitative assessments of the mean RNFL and GC-IPL thickness. SD-OCT images can also be assessed qualitatively. This includes the transverse axial scan of the ONH and peripapillary tissues to evaluate shape deformations, pseudopapilledema with and without drusen, peripapillary and macular edema, inflammatory signs, maculopathies, and folds. En face SD-OCT is a sensitive way of screening for PPW and outer retinal creases, both distinctive signs of optic disc edema. The circular tomogram that accompanies the mean RNFL thickness report identifies segmentation failures and transversely oriented folds in the choroid or retina. Finally, examination of the ONH in adduction may expose folds and deformations that may not be evident in primary position in patients with papilledema and other causes of optic disc edema. The interpretation of the SD-OCT depends on an understanding of its limitations and artifacts in the context of the overall history and clinical findings.

STATEMENT OF AUTHORSHIP

Category 1: a. Conception and design: P. A. Sibony, M. J. Kupersmith, and R. H. Kardon; b. Acquisition of data: P. A. Sibony, M. J. Kupersmith, and R. H. Kardon; c. Analysis and interpretation of data: P. A. Sibony, M. J. Kupersmith, and R. H. Kardon. Category 2: a. Drafting the manuscript: P. A. Sibony, M. J. Kupersmith, and R. H. Kardon; b. Revising it for intellectual content: P. A. Sibony, M. J. Kupersmith, and R. H. Kardon. Category 3: a. Final approval of the completed manuscript: P. A. Sibony, M. J. Kupersmith, and R. H. Kardon.
